# Effects of paternal methionine supplementation on sperm DNA methylation and embryo transcriptome in sheep

**DOI:** 10.1093/eep/dvac029

**Published:** 2022-12-23

**Authors:** Jessica Townsend, Camila U Braz, Todd Taylor, Hasan Khatib

**Affiliations:** Department of Animal and Dairy Sciences, University of Wisconsin-Madison, 1675 Observatory Dr., Madison, WI 53706, USA; Department of Animal and Dairy Sciences, University of Wisconsin-Madison, 1675 Observatory Dr., Madison, WI 53706, USA; Department of Animal and Dairy Sciences, University of Wisconsin-Madison, 1675 Observatory Dr., Madison, WI 53706, USA; Department of Animal and Dairy Sciences, University of Wisconsin-Madison, 1675 Observatory Dr., Madison, WI 53706, USA

**Keywords:** DNA methylation, fetal programming, paternal effect, nutritional epigenetics, embryo transcriptomics

## Abstract

Environmental effects on gene expression and offspring development can be mediated by epigenetic modifications. It is well established that maternal diet influences DNA methylation patterns and phenotypes in the offspring; however, the epigenetic effects of paternal diet on developing offspring warrants further investigation. Here, we examined how a prepubertal methionine-enriched paternal diet affected sperm DNA methylation and its subsequent effects on embryo gene expression. Three treatment and three control rams were bred to seven ewes, and blastocysts were flushed for RNA extraction. Semen was collected from all rams and submitted for reduced representation bisulfite sequencing analysis. In total, 166 differentially methylated cytosines were identified in the sperm from treatment versus control rams. Nine genes were found to be differentially expressed in embryos produced from treatment versus control rams, and seven differentially methylated cytosines in the sperm were found to be highly correlated with gene expression in the embryos. Our results demonstrate that sperm methylation differences induced by diet may influence fetal programming.

## Introduction

The importance of an optimal uterine environment and proper maternal nutritional requirements has been well established. Epidemiological studies demonstrate a strong connection between maternal health during pregnancy and diseases in the offspring. Studies have shown that there is an increased risk of childhood obesity in offspring born to diabetic mothers [1] and an increased risk of high blood pressure in offspring exposed to high protein intake during pregnancy [2], and high sugar intake during pregnancy has been associated with childhood atopy and asthma [3]. Anway et al. exposed gestating female rats to the endocrine disruptors such as vinclozolin or methoxychlor, which led to decreased spermatogenic capacity and increased male infertility from the F1 to F4 generation [4]. These connections between maternal and offspring health form the premise of the Developmental Origins of Health and Disease hypothesis, which outlines how environmental insults during critical periods of development lead to epigenetic modifications, changes in gene expression, and overall phenotypic differences [5].

While the maternal effects on offspring are fairly well understood, the paternal effects on offspring development need further evaluation. Studies have shown that obese male mice produce offspring with insulin resistance, glucose intolerance, and increased adiposity [6]. Interestingly, adult offspring from male mice fed a low protein diet also display impaired glucose tolerance, cardiovascular dysfunction, and increased adiposity [7]. Similar to the Developmental Origins of Health and Disease hypothesis, these connections between paternal health and offspring health form the Paternal Origins of Health and Disease (POHaD) hypothesis [8].

Years of research have demonstrated that paternal environmental insults can affect the phenotype of offspring; however, it is crucial to further investigate the mechanisms by which these insults are seemingly inherited. Jirtle and Skinner demonstrated that environmentally induced epigenetic changes such as DNA methylation, histone modifications, and noncoding RNAs could be transferred through the germline to the next generation [9]. When looking at sperm from obese males, they display DNA methylation differences compared to lean males [10]. DNA methylation differences in imprinted genes in offspring from obese fathers have also been observed [11]. In mice, male obesity led to alterations in microRNA content and a reduction in DNA methylation in sperm [6]. Undernutrition has also been associated with sperm hypomethylation [12]. In another study, Kubsad et al. exposed gestating rats to the herbicide known as Roundup and identified differentially methylated regions (DMRs) in the sperm of treated versus control males from three subsequent generations [13].

Most paternal epigenetic inheritance research has used mouse and rat models, while livestock species have been largely ignored, leading our laboratory to investigate the inheritance of DNA methylation throughout several generations in sheep. Braz et al. [14] and Gross et al. [15] found that supplementing methionine, a methyl donor, to prepubertal rams led to differentially methylated cytosines (DMCs) in the sperm of the F0 generation, some of which were inherited by the F1 and F2 generations. Phenotypic traits such as scrotal circumference and loin muscle depth were also influenced by the paternal diet and inherited by subsequent generations [14, 15].

These studies have shown that epigenetic marks caused by environmental changes are heritable to future generations. However, the embryonic stage has not been evaluated regarding paternal nutritional changes. Previous research demonstrated that adding dietary methionine to cows at the preimplantation stage of embryo development altered the expression of 276 genes in collected blastocysts [16]. Other studies reported that fetuses produced from sheep fed either hay or corn during the late gestation displayed differential methylation and gene expression in muscle and adipose tissues [17–19]. Therefore, we hypothesized that the exposure of prepubertal males to a methyl donor, methionine, would alter sperm DNA methylation, leading to altered embryo gene expression.

## Results

### Effects of Diet on Methylation Patterns in Sperm

**Figure 1:**
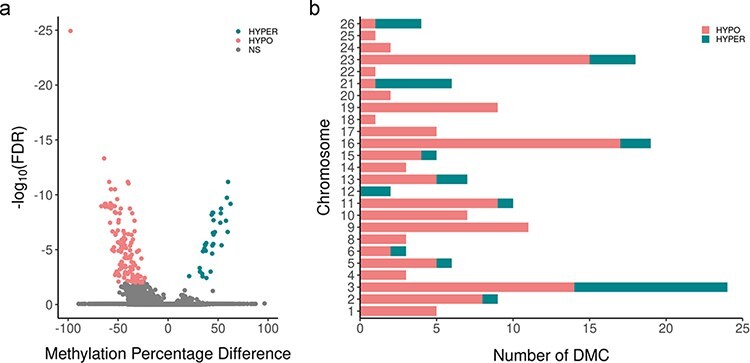
(a) The volcano plot of DMC analysis. DMCs were defined as those with a methylation difference (*x*-axis) >20% between treatment and control animals with an FDR of 0.01 (*y*-axis) as threshold values. Pink and blue dots represent DMCs hypomethylated and hypermethylated in the methionine-treated group compared to the control group, respectively. (b) DMC chromosomal distribution from sperm. DMCs were distributed throughout 25 (of 26 total) autosomal chromosomes. Pink shading represents DMCs that were hypomethylated, and blue shading represents DMCs that were hypermethylated in methionine-treated versus control rams

**Figure 2:**
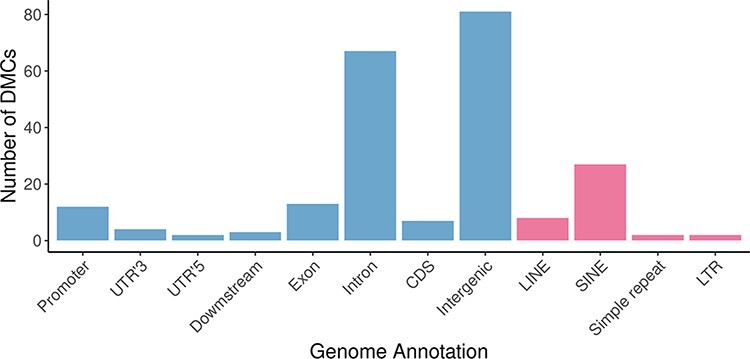
Location of DMCs between sperm from methionine-treated and control rams throughout the genome

To assess if the methionine supplementation affected DNA methylation patterns in the sperm, we performed reduced representation bisulfite sequencing (RRBS) analysis for the three methionine-treated rams and the three control rams. In total, 1 850 625 Cs at CpG context were found, of which 166 were differentially methylated between the two groups [DMCs; false discovery rate (FDR) <0.01; methylation difference >20%]. A total of 45 DMRs were also identified, which contained 11 overlapping DMCs (Supplementary Table S1). DMCs spanning 25 chromosomes were identified, with 39 DMCs displaying a methylation difference of >50% (Fig. 1). Approximately half of the DMCs mapped to intergenic regions, while ∼40% were located in intronic regions, 8% localized to promoter regions and exons, and <5% resided in 3ʹ and 5ʹ untranslated regions. About 23% of the DMCs mapped to repeat sequences, including the transposable elements long interspersed nuclear elements, short interspersed nuclear elements, and long terminal repeat (Fig. 2). Of the 166 DMCs, 134 were hypomethylated, and 32 were hypermethylated in the treatment group compared to the control group.

### Differentially Expressed Genes in Embryos

To evaluate whether the addition of methionine to the paternal diet affects gene expression in the embryos produced from the breeding, we performed RNA sequencing from six control pools and six treatment pools of five embryos each. No morphological differences were found between the two embryo groups. Of the 16 979 expressed genes analyzed, nine genes were differentially expressed between embryos from the two groups (FDR <0.05), of which six genes were downregulated and three were upregulated in the embryos from treatment sires compared to those from control sires (Table 1). This result suggests that paternal diet may affect embryo transcriptomics, likely through epigenetic mechanisms such as DNA methylation and other paternal factors such as histone modifications and noncoding RNAs.

**Table 1:** Differentially expressed genes between embryos produced from treatment versus control rams

Gene	logFC	logCPM	*P*-value	FDR	Function
*LOC105604880*	−3.762	5.13	5.54e-07	9.4e-03	Uncharacterized
*HRC*	−1.846	4.51	5.99e-06	2.94e-02	Encodes a luminal sarcoplasmic reticulum protein; may regulate sarcoplasmic reticulum calcium release [83]
*LOC114111604*	−2.500	4.57	6.93e-06	2.94e-02	Uncharacterized
*UNC93A*	−1.563	4.88	6.60e-06	2.94e-02	Located in plasma membrane; expressed in brain and peripheral tissues [84]
*TRAM1L1*	4.146	6.89	9.31e-06	3.16e-02	Largely unknown; may have some neuronal functions [85]
*C20H6orf136*	1.405	5.43	1.85e-05	3.49e-02	Uncharacterized
*CERS1*	2.277	4.85	1.77e-05	3.49e-02	Encodes a ceramide synthase enzyme that synthesizes ceramide in the brain and neurons [68]
*DENND6B*	−5.244	7.24	1.41e-05	3.49e-02	Enables guanyl-nucleotide exchange factor activity; involved in the endocytic recycling pathway and the stability of adherens junction in migrating cells [86]
*GGT7*	−3.828	7.74	1.56e-05	3.49e-02	Member of a gene family that encodes enzymes involved in the metabolism of glutathione and the transpeptidation of amino acids [69]

logFC = Log-fold change.

### Correlation between DMCs Detected in Sperm and Embryo Gene Expression

To assess if sire sperm methylation changes as a response to methionine supplementation are related to gene expression levels in the embryos, we performed a correlation analysis between sperm DMCs and embryo gene expression. A total of seven DMCs found in sperm were highly correlated (*r* > 0.7) and statistically significant (*P* < 0.10) with gene expression levels in the embryos (Table 2), suggesting that the methyl marks influenced by the paternal diet may possibly be transmitted to the embryo and affect the transcription efficiency.

**Table 2:** Correlation between DMCs in sperm and gene expression in embryos

Gene	Location of C^a^	Methylation status of sperm	Correlation value	*P*-value	Function
*ADNP2*	23:592 007 Exon	Hypomethylated	−0.949	0.004	Associated with cell survival, protect neuron like cells, and may play a role in the SWI/SNF chromatin remodeling complex [75]
*CARS*	21:50 425 765 Intron	Hypomethylated	−0.839	0.0366	Encodes a class 1 aminoacyl-tRNA synthetase; located near an important tumor suppressor gene [87]
*UBE3D*	8:11 335 792 Intron	Hypomethylated	0.824	0.044	Enables cyclin-binding activity and ubiquitin protein ligase activity [82]
*LOC101103023*	21:39 594 253 Promoter	Hypermethylated	0.820	0.046	Uncharacterized
*GALNT13*	2:167 665 401 Intron	Hypomethylated	−0.762	0.078	Initiates O-linked glycosylation of mucins [81]
*UBE3D*	8:11 335 967 Intron	Hypomethylated	0.759	0.080	Enables cyclin-binding activity and ubiquitin protein ligase activity [82]
*AK8*	3:4 288 286 Intron	Hypermethylated	−0.757	0.0817	Activated during spermatogenesis and plays role in flagellar biosynthesis; may play role in meeting energy requirements of other motile cilia [64, 65]

a Chromosome: position in base pairs.

## Discussion

### Effects of Diet on Methylation Patterns in Sperm

For decades, human and animal studies have aimed at understanding maternal programming, while the effects of paternal programming on offspring health have largely been overlooked. In recent years, more emphasis has been placed on evaluating genetic and epigenetic factors that are contributed by the sperm or even the seminal fluid to the offspring [8]. Our previous studies have shown that methionine supplementation to prepubertal rams affects DNA methylation patterns in the sperm of the F0 generation [14, 15]. Furthermore, these methylation signatures were passed onto the next two generations, demonstrating that epigenetic marks are heritable and may play vital roles in offspring development [14, 15]. Consistent with these findings, the present study found 166 DMCs in sperm from methionine-treated rams versus control rams.

Of the 166 DMCs, 134 were hypomethylated, while 32 were hypermethylated in treatment versus control animals. While methionine and other methyl donors have been shown to increase DNA methylation in various studies [20, 33–35], there are also numerous accounts of methionine leading to a decrease in DNA methylation. Gross et al. also supplemented prepubertal rams with methionine and found 426 hypomethylated DMCs and 398 hypermethylated DMCs in treatment versus control rams [15]. Pogribny et al. found that feeding methionine to middle-aged rats for 14 days led to substantial DNA hypomethylation and increased plasma homocysteine levels [36]. Another group reported that feeding methionine to peripartal cows resulted in lower global hepatic DNA methylation, yet also found the promoter region of *PPARA* hypermethylated, resembling the pattern shown in our results [37]. The maintenance and establishment of methyl marks depend on the one-carbon cycle where methionine is converted into S-adenosylmethionine (SAM), which then acts as a methyl donor for methyltransferases. After donating its methyl group, SAM is converted into S-adenosylhomocysteine (SAH), and then a hydrolysis reaction converts SAH into homocysteine [38]. SAH however is an inhibitor of most methyltransferases, and therefore, the ratio of SAM to SAH is essential for transmethylation potential [39]. Importantly, SAM also functions as an allosteric inhibitor of 5,10-methylene-tetrahydrofolate reductase found in the folate cycle, and its product, 5-methyl-tetrahydrofolate, serves as a ligand of glycine-N-methyltransferase (GNMT) that upon binding inhibits its enzymatic activity [40–42]. GNMT is a key protein in regulating the SAM:SAH ratio. When there is excess methionine present in the cycle, this leads to an increase in SAM, which inhibits 5,10-methylene-tetrahydrofolate reductase and therefore reduces the amount of 5-methyl-tetrahydrofolate. Consequently, the inhibition of GNMT is alleviated, enabling the excess methyl groups from methionine to be disposed of as sarcosine rather than added to DNA methyltransferases for DNA methylation [43, 44]. Interestingly, several studies supplementing methionine have found increased SAH concentration, while SAM levels stayed the same [35, 45]. Since SAH is an inhibitor of DNMT activity [39], it is possible that the supplementation of methionine provided to the treatment group in our study would have led to hypomethylation of cytosine compared to the control group.

The intragenic DMCs identified in this study are of particular interest as methylation in introns may be important for regulating alternative splicing [46], while methylation in promoters, exons, and introns has been shown to alter gene expression through transcriptional silencing and alterations in chromatin structure [47–50].

Interestingly, 23% of the DMCs influenced by the paternal diet were mapped to repeat sequences, including transposable elements. Transposable elements make up half of most mammalian genomes [51] and must be tightly regulated through DNA methylation to prevent potentially detrimental transpositions [52]. It is important to note that during the zygotic stage, epigenetic reprogramming occurs, and epigenetic marks are mostly erased [53]. However, imprinted regions and transposable elements are known to resist epigenetic reprogramming. Retaining DNA methylation in transposable elements is essential as it prevents their mobilization through the genome, which helps to maintain genomic stability [52]. DNA methylation changes in transposable elements have been associated with obesity [35]. In addition, the activation of some transposable elements has been linked to congenital diseases, infertility, and cancer [54]. Maternal supplementation of methyl donors to pregnant mice affected coat color from yellow to brown and obesity in subsequent generations [55]. These phenotypes were associated with DNA methylation changes in a transposable element upstream of the Agouti gene. Our study introduced a slight increase in dietary methionine, which led to substantial changes in DNA methylation within repeat sequences, indicating that transposable elements are vulnerable to environmental changes. Thus, nutritional inputs could affect phenotypic outcomes over generations, given that transposable elements escape the epigenetic reprogramming events.

Several genes harboring DMCs have been associated with anatomical structure morphogenesis, including *ZNRF3*, *FOXF2*, *CHST11*, and *CCBE1*. Harris et al. [56] found that *ZNRF3* is a Wingless-related integration site signaling antagonist required for testis determination in mice. Male mice lacking *ZNRF3* display complete or partial gonadal sex reversal [56]. *FOXF2*, on the other hand, has been shown to have crucial effects on a plethora of embryonic developmental processes, including development of the lip, tooth, tongue, eye, cochlea, palate, cerebrum, gastrointestinal tract, bronchus, lung, tendon, diaphragm, cartilage, and heart, mainly through its role in cell differentiation and controlled proliferation [57]. *CHST11* is suggested to play an important role in cartilage and bone development. Interestingly, mice homozygous for a loss-of-function mutation in the *CHST11* gene die shortly after birth and exhibit severe chondrodysplasia [58, 59]. Lastly, Bos et al. [60] found *CCBE1* to be essential for lymphangiogenesis, with knockout mice dying prenatally and lacking all lymphatic vessels.

Additional genes containing DMCs have previously been found to have roles in nervous system development, including *WWOX*, *JADE2*, *NF2*, *TBCD*, *SLIT3*, *CNTF*, *DAB2IP*, *CAMSAP2*, *AK8*, *FSTL4*, and *PLXND1*. During mouse embryonic development, *WWOX* is highly expressed in the neural crest-derived structures, indicating a possible role of this gene in neuronal differentiation and maturation [61]. *WWOX* knockout mice experience cerebral malformations, neuronal disorganization and heterotopia, and defective cerebellar midline fusion, which mimic many of the abnormalities seen in humans having loss-of-function mutations in both alleles of *WWOX* [61]. McClatchey et al. [44] reported that the *NF2* gene encodes for the protein known as merlin in Schwann cells that insulate nerves. *NF2-*deficient mouse embryos arrest at E6.5-E7.0 and display abnormal extraembryonic structures and failed gastrulation [62].

The fact that so many genes harboring DMCs with known functions in the nervous system were found in the sperm is rather perplexing; however, all the above nervous system genes, apart from *WWOX*, have shown similar proteins between the testis and the brain tissues [63]. Notably, adenylate kinase 8 (*AK8*) is activated during spermatogenesis, and the protein production occurs during flagellar biogenesis, with the protein being localized to the flagellum and the acrosomal region of cauda epididymal sperm [64]. It is thought that *AK8* may allow for buffering of Adenosine triphosphate (ATP) among the axoneme and flagellum [64]. This ATP buffering is essential for the sperm cell as the limited amount of cytoplasm and compartmentalization of glycolysis and oxidative phosphorylation reduce the distribution of ATP [64]. The buffering of ATP through *AK8* may also be crucial in meeting the high-energy demands of other motile cilia, such as those associated with ependymal cells [65]. *AK8* knockout mice developed mild-to-moderate hydrocephalus [65], which can be caused by defective cilia blocking the flow of cerebrospinal fluid [66].

### Differentially Expressed Genes in Embryos

To examine the effect of paternal diet on embryo transcriptomics, we performed RNA sequencing from the embryos produced from methionine-treated and control rams. With the understanding that epigenetic marks are heritable throughout generations [14], it is not surprising that differential methylation patterns in the sperm could lead to differential gene expression in the embryos, as DNA methylation is well known to regulate transcription efficiency [67]. Most of the nine differentially expressed genes found in embryos are either uncharacterized or very little is known about their function. Of interest is ceramide synthase 1 (*CERS1*), which was found to be upregulated in embryos produced from methionine-supplemented sires. This gene encodes a ceramide synthase enzyme that catalyzes the synthesis of C18 ceramide, the hydrophobic component of sphingolipids, and is mainly expressed in the brain. *CERS1-*derived ceramide is essential in the regulation of cerebellar development, with CERS1-deficient mice displaying morphological abnormalities of the cerebellum, including smaller size, foliation defects, and neuronal apoptosis [68]. It was also found that C18 ceramide is an essential precursor for the biosynthesis of gangliosides in the cerebellum and forebrain [68].

Another interesting gene found to be downregulated in embryos produced from methionine-treated rams is the gamma-glutamyl transferase 7 (*GGT7*), which encodes an enzyme involved in glutathione metabolism [69]. Glutathione is critical for many cellular processes, including cell proliferation, regulation of gene expression, DNA and protein synthesis, protein folding, signal transduction, and regulation of immune response [70–72]. Glutathione is also likely the central component of redox regulation in embryogenesis and organogenesis [73], warranting further investigation into the role of *GGT7* in embryo and fetal development. It is important to note that while some of these genes may have roles in embryogenesis, it is likely that these differentially expressed genes may not cause any effects until further into development, or even adulthood, as outlined by the POHaD hypothesis [8]. This hypothesis describes how epigenetic sperm modifications can affect offspring health status, allowing us to hypothesize that the methionine supplementation to the rams leads to possible incidents of fetal programming in the offspring.

### Correlation between DMCs in Sperm and Embryo Gene Expression

The sperm epigenome can transfer preconceptual environmentally derived changes from the father to the offspring through DNA methylation, histone modifications, or small noncoding RNAs [74]. Therefore, we evaluated the correlation between methylation levels in the sperm and gene expression levels in their respective embryos to gain insight into one possible mechanism of gene regulation. While sperm DNA methylation and embryonic gene expression differences did not appear to affect blastocyst morphology or early developmental capacity, it is plausible to surmise that these genes may play roles in the fetal or even postnatal stages of life, as described by the POHaD hypothesis. For example, of the seven genes we found with a strong correlation between sperm DNA methylation and gene expression in embryos, one of particular interest is the Homeobox Protein 2 (ADNP) Homeobox 2 (*ADNP2*) gene, which has been found to be highly expressed in the embryonic mouse brain, and its expression is sustained through adulthood [75]. *ADNP2*’s homolog, *ADNP*, is essential for brain formation and function [76, 77], neurite outgrowth [78], and glial-derived neuroprotection, and a complete deficiency is considered embryonic lethal [79]. Similar neuron-protecting functions have been found for *ADNP2* although they have not been deeply explored [75]. Knockdown of *ADNP2* in zebra fish led to cardiac edema and lack of blood cells in early embryos, and death occurred for most embryos during Week 1, demonstrating that this gene is essential for primitive erythropoiesis [80]. *ADNP* and *ADNP2* have also been shown to bind with Brg1 and function as part of the SWItch/Sucrose Non-Fermentable (SWI/SNF) chromatin remodeling complex [77, 80]. Another gene that seems to support the notion of paternal fetal programming is the Polypeptide N-Acetylgalactosaminyltransferase 13 (*GALNT13*) gene. This gene has been found to be highly upregulated during early neurogenesis in mouse embryonic brains, and a knockout of this gene suppressed neural induction and neuronal differentiation [81]. Furthermore, supporting the idea that differential methylation may affect later development is the activity of the ubiquitin protein ligase E3D (*UBE3D*) gene. In a zebra fish embryo model, low expression of *UBE3D* promotes oxidative damage and inflammatory reactions, and its knockdown leads to delayed eye development, reduced eye size, apoptosis, and overall retinal degeneration [82]. Other genes, such as *CARS* and *LOC101103023*, should be further analyzed for potential roles in the early development.

Future studies should replicate this experiment with a larger population of animals and include DNA methylation analysis and RNA sequencing of embryos. The utilization of whole genome bisulfite sequencing would also be beneficial in capturing methyl marks not detected by RRBS. Additionally, further work should be conducted to evaluate gene expression and phenotypic differences of grown F1 animals.

## Conclusions

This study aims to understand further how environmentally induced epigenetic marks can affect embryo transcriptomics and potentially affect offspring phenotypes. The results of this study validate our previous findings that providing rumen-protected methionine (RPM) supplementation to prepubertal rams leads to changes in DNA methylation in the sperm. These methyl marks are highly correlated with the varying gene expression levels observed in the embryos. The current results reveal insights into the initial stages of paternal fetal programming and help us to better understand the far-reaching impact that paternal diet has on embryo development.

## Material and Methods

All procedures involving animals were approved by the Institutional Animal Care and Use Committee of the University of Wisconsin-Madison (Protocol ID: A006488).

### Supplementation of RPM in Diets of F0 Rams

Eight Polypay rams were selected, with four randomly assigned to the control and four to the treatment diet. The control diet was a general basal diet [15], and the treatment was the control diet plus a 0.22% (1.5 g) added top-dress of RPM (RPM Smartamine; Adisseo, Alpharetta, GA, USA). Methionine was selected for supplementation as it is a known methyl donor and has been shown to induce changes in DNA methylation [20]. Rams were separated into individual pens and fed 0.45 kg of their respective diet twice daily for 77 days. The average age of the rams at the beginning and at the end of the trial were 88 and 165 days (standard deviation of 4.8 days), respectively. Between feedings, the rams were group housed with free access to forage, water, and a total of 1 kg of the basal diet.

### Semen Collection

It has been established that epigenetic marks can be passed through the germline to the next generation [9]; therefore, semen was collected 1 month after the end of the methionine supplementation period for DNA methylation analysis via electroejaculation using the Lane Pulsator IV (Lane Manufacturing Inc., Denver, CO, USA) as described by Gross et al. [15]. Briefly, the probe was inserted into the rectum, and ejaculate was collected in a 15-ml conical vial. A prewarmed (37°C) semen extender was added to the semen, and motility and sperm concentration were evaluated using iSperm (Aidmics Biotechnology Co., Ltd, Taiwan). Only samples meeting the criterion of 50 million sperm per ejaculate with >10% motility were used for further analysis, as this is a standard indication of puberty [21]. One treatment and one control ram did not pass this sperm quality control and therefore were removed from the study. The remaining semen was washed twice with phosphate-buffered saline (PBS), pelleted by centrifugation, and stored in RNAlater (Thermo Fisher Scientific, Waltham, MA, USA) at −20°C.

### Reduced Representation Bisulfite Sequencing

Genomic DNA was extracted from sperm using the Quick-DNA Miniprep Plus Kit (Zymo Research, Irvine, CA, USA). Briefly, 100 µl of sperm in RNAlater was washed with 200 µl of PBS and centrifuged for 3 minutes at 20 000 g to pellet the sperm cells. The supernatant was then removed, and the pellet was washed once more and centrifuged again. After the removal of the supernatant, the pellet was resuspended in 200 µl of somatic cell lysis buffer for 4 minutes on ice [22]. Following incubation, the sperm cells were centrifuged for another 4 minutes at 20 000 g, the supernatant was removed, and the pellet was resuspended in 200 µl of PBS and vortexed before adding 200 µl of lysis buffer and 20 µl of Proteinase K. The samples were incubated overnight at 55°C, and the extraction proceeded per the manufacturer’s protocol. DNA quality and quantity were determined by Qubit Fluorometer (Thermo Fisher Scientific, Wilmington, DE) and electrophoresis before RRBS.

RRBS was performed at Roy J. Carver Biotechnology Center at the University of Illinois, Urbana-Champaign. The six samples, containing 1250 ng of DNA each, were bisulfite treated using the EZ-DNA Methylation Lightning Kit (Zymo Research, Irvine, CA). Then, libraries were prepared using the Ovation RRBS Methyl-Seq Kit (Tecan Genomics, Redwood City, CA). The libraries were then pooled, quantified by quantitative PCR (qPCR), and sequenced on one SP lane on an Illumina NovaSeq 6000 sequencing platform, producing 100-bp single-end reads (Illumina, San Diego, CA, USA) and 6-bp unique molecular identifiers. The generated FASTQ files were demultiplexed using bcl2fastq Conversion Software (v2.20, Illumina). On average, 56.6 M reads were generated, varying from 52.9 to 59.4 M. FastQC software (v.0.11.8, https://www.bioinformatics.babraham.ac.uk/projects/fastqc/) was used to check the quality of the reads in each sample. Unique molecular identifiers were added to the reads using “append_barcodes.py” from the NuGen to remove polymerase chain reaction (PCR) duplicates. Then, TrimGalore (v.0.6.5, https://www.bioinformatics.babraham.ac.uk/projects/trim_galore/) was used to discard low-quality reads and bases and adapter sequences.

### RRBS Alignment and DNA Methylation Analysis

Cleaned reads were aligned using bowtie2 of the Bismark software (v.0.22.3) [22] to the sheep reference genome, Oar_rambouillet_v.1.0. The bisulfite conversation rate and the C methylated in CG context were, on average, 99.5% and 36.3%, respectively. The percentage of alignment per sample varied from 54.0% to 58.5%, with an average of 55.8%. Then, the option “-barcode” of the “deduplicate_bismark” function was used to filter out duplicate reads and PCR errors. The CG methylation calling was performed using the “bismark_methylation_extractor” function [22].

DMC and DMR analyses between the control and the treatment groups were performed using a logistic regression model with overdispersion correction implemented in the R package “methylKit” [23], as follows: $\log \left( {{\mathrm{M}}{{\mathrm{P}}_i}/1 - {\mathrm{M}}{{\mathrm{P}}_i}} \right) = {\beta _0} + {\beta _1}{\mathrm{ag}}{{\mathrm{e}}_i} + {\beta _2}{\mathrm{die}}{{\mathrm{t}}_i}$, where ${\mathrm{M}}{{\mathrm{P}}_i}$ is the methylation proportion at a given CG site per region for sample $i$; ${\beta _0}$ is the intercept; ${\beta _1}$ and ${\beta _2}$ are the fixed effects of age (covariate) and diet, respectively; and ${\mathrm{ag}}{{\mathrm{e}}_i}$ and ${\mathrm{die}}{{\mathrm{t}}_i}$ are vectors that specify the age and treatment corresponding to sample $i$. For each CG site per region, the logistic regression model was independently fitted to evaluate the alternative hypothesis $\left( {{H_1}:\,{\beta _2} \ne 0} \right)$ against the null hypothesis $\left( {{H_0}:\,{\beta _2} = 0} \right)$. Only cytosines with a minimum of 10 counts among all samples were analyzed. CG regions were generated with a window size of 200 bp, step size of 50 bp, and containing at least three CG sites, using the tilling window approach from methylKit [23] DMR analysis was performed in order to verify if DMCs detected in this study aggregate with multiple CG sites. The criteria to define significant DMCs/DMRs were as follows: methylation difference >20% between the two groups and FDR <0.01. DMCs were considered hypermethylated or hypomethylated when presenting higher or lower methylation, respectively, in the treatment animals compared to the control animals. DMCs were annotated for repetitive element regions downloaded from the University of California, Santa Cruz database [24]. In addition, DMC/DMR positions were overlapped with exon, intron, promoter, upstream and downstream gene regions (200 bp), and intergenic regions. Promoter regions were defined as 10 kb upstream of genes transcription start sites. The “ggplot2” R package was used to generate the figures [25].

### Superovulation and Synchronization Protocols

To produce the maximum number of embryos for transcriptomic analysis, eight randomly selected Polypay ewes underwent estrus synchronization and superovulation prior to breeding. Briefly, a controlled internal drug release (CIDR) was vaginally inserted into each ewe on Day 0, and then, on Day 7, a new CIDR replaced the original one, and 1.0 cc of prostaglandin (EstroPLAN) was administered. Beginning on Day 12, Folltropin was injected twice daily over 4 days. The CIDR was removed on Day 14.5, and 1.5 cc of PG600 was given. On Day 16, each ram was randomly introduced into a pen with one or two ewes. Rams were affixed with marking harnesses to confirm successful breeding. The breeding occurred ∼2 months after the end of the methionine supplementation period.

### Embryo Flushes

Embryo collections were performed by GenOvations (Lodi, WI, USA) via laparotomy 8 days after rams were introduced to the ewes. The ewes were fasted for 24 hours before surgery. Ewes first received 2–4 mg/kg of propofol, followed by inhalation of the anesthetic, isoflurane. Once intubated, the ewes were placed in a Trendelenburg position, and a ∼1.5 cm incision was made in their abdomen. The laparoscope was inserted through the incision to visualize the uterus, and the incision was then expanded to ∼7 cm through the subcutaneous tissue, abdominal musculature, and peritoneum. The uterine horns were then exposed, and a 20-G Foley catheter was inserted into the distal third of the uterine horn. Saline (50 ml) was rinsed through each uterine horn and collected through the Foley Probe into a filtered Petri dish. The uterine horns were then repositioned in the abdomen, and the musculature and skin were sutured [26]. Immediately following the embryo flush, blastocysts were washed in PBS, evaluated using the International Embryo Transfer Society grading system, placed into RNAlater, and stored at −80°C. A total of 36 grade 1 (excellent) embryos were collected from ewes mated with control rams, and from ewes mated with treatment rams, 35 grade 1 (excellent) and three grade 2 (good) embryos were collected. All embryos used for downstream analysis were stage 5 (early blastocyst) through stage 8 (hatched blastocyst) and displayed a balanced distribution of stages throughout the groups.

### RNA Sequencing and Differential Expression Analysis of Embryos

**Figure 3:**
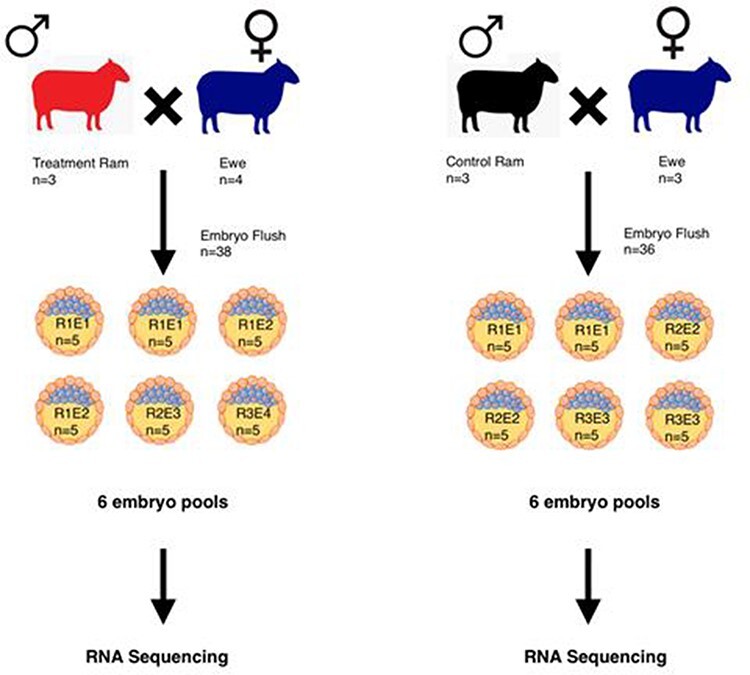
The experimental design outlining the number of embryos collected from each mating. R-number designates the ram that was used for the breeding, and E-number identifies the ewe that the embryos were collected from

Embryos were grouped into 12 pools based on sire and dam information, of which six pools represent the treatment group and six pools the control group, with each pool containing five embryos (Fig. 3). Total RNA was extracted from embryos using the RNAqueous-Micro Kit (Ambion, Austin, TX, USA). DNA removal and whole transcriptome amplification were performed using the REPLI-g WTA Single Cell Kit (Qiagen, Germantown, MD, USA).

RNA sequencing for the 12 pooled samples was performed using Illumina NovaSeq 6000 (Illumina, San Diego, CA, USA), which generated 39.1 M 100-bp single-end reads, on average, varying from 33.4 to 47.9 M. The FASTQ files were demultiplexed with the bcl2fastq Conversion Software (v.2.20) and checked for reads quality with the FastQC software (https://www.bioinformatics.babraham.ac.uk/projects/fastqc/). To trim low-quality reads and bases and remove adapter sequences, Trimmomatic was used [27] in each sample. The trimmed reads were then aligned to the sheep reference genome (Oar_rambouillet_v1.0) using STAR [28] including the “--quantMode GeneCounts” option to obtain gene counts. In total, 16 979 expressed genes, with at least 15 counts in more than three samples, were considered for further analysis. Gene counts were normalized based on the trimmed mean of M-values method using the “edgeR” R package [29]. Differential expression analysis between the treatment and control groups was carried out based on a negative binomial generalized linear model implemented in the “edgeR” package, as follows: $\log {\mu _{gi}} = {\beta _g}{x_i} + \log {N_i}$, where ${\mu _{gi}}$ is the product of the relative abundance of the gene $g$ of the sample $i$ and the effective library size of the sample $i$; ${x_i}$ is a vector that specifies the treatment applied to sample $i$; ${\beta _g}$ is a vector of regression coefficients by which the treatment effect is mediated for gene $g$; and ${N_i}$ is the effective library size of the sample $i$. For more details about the calculation of the effective library sizes, please see [30]. Genewise tests were conducted by computing likelihood-ratio statistics to compare the null hypothesis against $\left( {{H_o}:\,{\beta _g} = 0} \right)$ the alternative hypothesis $\left( {{H_1}:\,{\beta _g} \ne 0} \right)$. The statistical tests were corrected for multiple testing, and only genes with FDR <0.05 were considered significant [31].

### Integration of DMC in Sperm and Gene Expression in Embryos

To evaluate if methylation levels in the sperm affected gene expression in the embryos, Pearson’s correlation (*r*) between methylation levels of DMCs in promoter regions and normalized expression values of the corresponding genes was calculated [32]. Correlation was considered significant for tests with *P*-value <0.10 and *r *>0.70 [32].

## Supplementary Material

dvac029_Supp

## Data Availability

RRBS and RNA sequencing data have been deposited in National Center for Biotechnology Information (NCBI)’s Gene Expression Omnibus (GEO) and are accessible through GEO Series accession number GSE215129.
